# Supplementary material for: Post-mortem Nasopharyngeal Microbiome Analysis of Zambian Infants With and Without Respiratory Syncytial Virus Disease: A Nested Case Control Study
Source: Pediatr Infect Dis J. Author manuscript; Available in PMC 2023 Sep 27. (PMC10348642; doi:10.1097/INF.0000000000003941)
Supplement: Supplemental Digital Content 4 [file NIHMS1888374-supplement-Supplemental_Digital_Content_4.pdf]

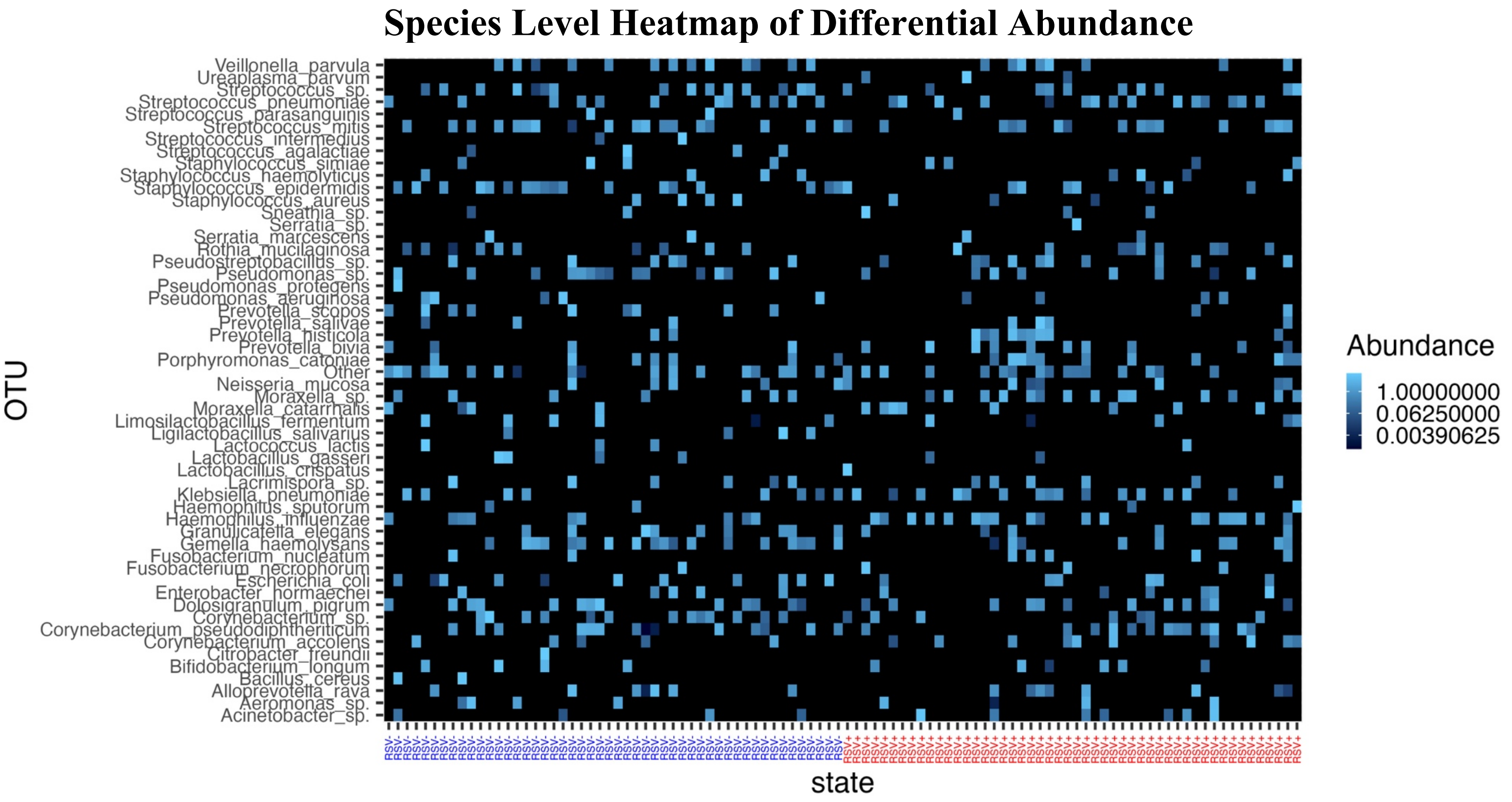

Figure, Supplemental Digital Content 4. Heatmap showing species differential abundance comparison. Note differences for *Corynebacterium accolens*, *Gemella haemolysans, Moraxella sp*., and *Prevotella scopos*.
