# Supplementary material for: Post-mortem Nasopharyngeal Microbiome Analysis of Zambian Infants With and Without Respiratory Syncytial Virus Disease: A Nested Case Control Study
Source: Pediatr Infect Dis J. Author manuscript; Available in PMC 2023 Sep 27. (PMC10348642; doi:10.1097/INF.0000000000003941)
Supplement: Supplemental Digital Content 2 [file NIHMS1888374-supplement-Supplemental_Digital_Content_2.pdf]

p-values and Adjusted p-values for Differential Abundance Analysis at Genus Level

**Genera**	**p-value**	**Adjusted p-value**
Acinetobacter	0.885	0.969
Aeromonas	0.447	0.838
Alloprevotella	0.829	0.969
Bacillus	0.747	0.969
Bifidobacterium	0.889	0.969
Citrobacter	0.973	0.978
Clostridium	0.978	0.978
Corynebacterium	0.871	0.969
Dolosigranulum	0.267	0.705
Enterobacter	0.124	0.446
Escherichia	0.117	0.446
Fusobacterium	0.274	0.705
Gemella	0.02**	0.24
Granulicatella	0.08	0.446
Haemophilus	0.059	0.425
Klebsiella	0.393	0.838
Lacrimispora	0.565	0.838
Lactobacillus	0.915	0.969
Lactococcus	0.519	0.838
Ligilactobacillus	0.141	0.461
Limosilactobacillus	0.582	0.838
Moraxella	0.006***	0.216
Mycoplasma	0.426	0.838
Neisseria	0.508	0.838
Other	0.357	0.838
Porphyromonas	0.11	0.446
Prevotella	0.551	0.838
Pseudomonas	0.707	0.969
Pseudostreptobacillus	0.877	0.969
Rothia	0.268	0.705
Serratia	0.486	0.838
Sneathia	0.547	0.838
Staphylococcus	0.018**	0.24
Streptococcus	0.051	0.425
Ureaplasma	0.793	0.969
Veillonella	0.099	0.446

Table, Supplemental Digital Content 2. Table of Wilcoxon rank sum test p-values testing RSV status differences with a Benjamini-Hochberg multiple testing adjustment. Significant values are marked with *** $p < 0.01$, ** $p < 0.03$, * $p < 0.05$.
