# Supplementary material for: Post-mortem Nasopharyngeal Microbiome Analysis of Zambian Infants With and Without Respiratory Syncytial Virus Disease: A Nested Case Control Study
Source: Pediatr Infect Dis J. Author manuscript; Available in PMC 2023 Sep 27. (PMC10348642; doi:10.1097/INF.0000000000003941)
Supplement: Supplemental Digital Content 5 [file NIHMS1888374-supplement-Supplemental_Digital_Content_5.pdf]

PICRUSt Pathway Abundance Analysis Significant Results

Pathway	Taxa w/pathway (per MetaCyc)	Adjusted p-value
6-hydroxymethyl-dihydropterin diphosphate biosynthesis III (Chlamydia)	*Chlamydia trachomatis*	0.029
8-amino-7-oxononanoate biosynthesis I	*Candidatus Nitrososphaera gargensis Ga9.2*<br>*Escherichia coli K-12 substr. MG1655*<br>*Francisella tularensis novicida U112*<br>***Haemophilus influenzae Rd KW20***<br>***Helicobacter pylori 26695***<br>***Mycobacterium tuberculosis H37Rv***<br>***Prochlorococcus marinus MIT 9211***	0.007
biotin biosynthesis I	*Escherichia coli K-12 substr. MG1655*<br>*Francisella tularensis novicida U112*<br>***Haemophilus influenzae Rd KW20***<br>***Helicobacter pylori 26695***<br>***Prochlorococcus marinus MIT 9211***	0.007
CMP-3-deoxy-D-manno-octulosonate biosynthesis I	*Arabidopsis thaliana col*<br>*Escherichia coli BL21(DE3)*<br>*Escherichia coli K-12 substr. MG1655*<br>*Zea mays*	0.003
flavin biosynthesis I (bacteria and plants)	*Arabidopsis thaliana col*<br>*Bacillus subtilis*<br>*Escherichia coli K-12 substr. MG1655*	0.029
fucose degradation	*Escherichia coli K-12 substr. MG1655*	0.045
Kdo transfer to lipid IVA III (Chlamydia)	*Chlamydia pneumoniae*<br>*Chlamydia psittaci*<br>*Chlamydia psittaci 6BC*	0.003
L-lysine biosynthesis I	*Arthrobacter globiformis*<br>*Azotobacter vinelandii*<br>*Bordetella pertussis*<br>*Corynebacterium glutamicum*<br>*Corynebacterium glutamicum ATCC 13032*<br>*Escherichia coli K-12 substr. MG1655*<br>***Haemophilus influenzae***<br>***Haemophilus influenzae Rd KW20***<br>*Helicobacter pylori*<br>*Mycobacterium tuberculosis*<br>*Mycobacterium tuberculosis H37Rv*<br>*Rhodospirillum rubrum*	0.034
L-lysine biosynthesis VI	*Arabidopsis thaliana col*<br>*Chlamydia trachomatis*	0.026

	Glycine max<br>*Methanocaldococcus jannaschii*<br>*Methanothermobacter thermautotrophicus*<br>*Nicotiana tabacum*<br>*Synechocystis*<br>*Zea mays*	
lipid IVA biosynthesis	*Caulobacter vibrioides NA1000*<br>*Escherichia coli K-12 substr. MG1655*	0.003
methylerythritol phosphate pathway I	*Brucella abortus*<br>*Escherichia coli K-12 substr. MG1655*	0.005
methylerythritol phosphate pathway II	*Arabidopsis thaliana col*<br>*Botryococcus braunii*<br>*Croton stellatopilosus*<br>*Plasmodium falciparum*<br>*Plasmodium falciparum HB3*<br>*Solanum lycopersicum*<br>*Thermosynechococcus vestitus BP-1*	0.005
mycolate biosynthesis	*Mycobacterium tuberculosis H37Rv*	0.023
NAD salvage pathway II	*Escherichia coli K-12 substr. MG1655*<br>*Salmonella enterica enterica serovar Typhimurium*	0.009
oleate biosynthesis IV (anaerobic)	*Aerococcus viridans*<br>*Cereibacter sphaeroides 2.4.1*<br>*Clostridium beijerinckii*	0.023
palmitate biosynthesis II (bacteria and plants)	*Arabidopsis thaliana col*<br>*Brassica napus*<br>*Escherichia coli K-12 substr. MG1655*<br>*Spinacia oleracea*	0.025
palmitoleate biosynthesis I (from (5Z)-dodec-5-enoate)	*Escherichia coli K-12 substr. MG1655*<br>*Helicobacter pylori 26695*	0.003
stearate biosynthesis II (bacteria and plants)	*Arabidopsis thaliana col*<br>*Brassica napus*<br>*Spinacia oleracea*	0.012
superpathway of (Kdo)2-lipid A biosynthesis	*Escherichia coli K-12 substr. MG1655*	0.028
superpathway of fatty acid biosynthesis initiation (E. coli)	*Escherichia coli K-12 substr. MG1655*	0.030

Table, Supplemental Digital Content 5. PICRUSt2 inferred pathways and associated species. These pathway abundances are all significantly different between RSV+ and RSV- samples. Species identified as being differentially expressed in RSV+ and RSV- decedents in our differential abundance analysis are bolded.
