# Supplementary material for: Post-mortem Nasopharyngeal Microbiome Analysis of Zambian Infants With and Without Respiratory Syncytial Virus Disease: A Nested Case Control Study
Source: Pediatr Infect Dis J. Author manuscript; Available in PMC 2023 Sep 27. (PMC10348642; doi:10.1097/INF.0000000000003941)
Supplement: Supplemental Digital Content 3 [file NIHMS1888374-supplement-Supplemental_Digital_Content_3.pdf]

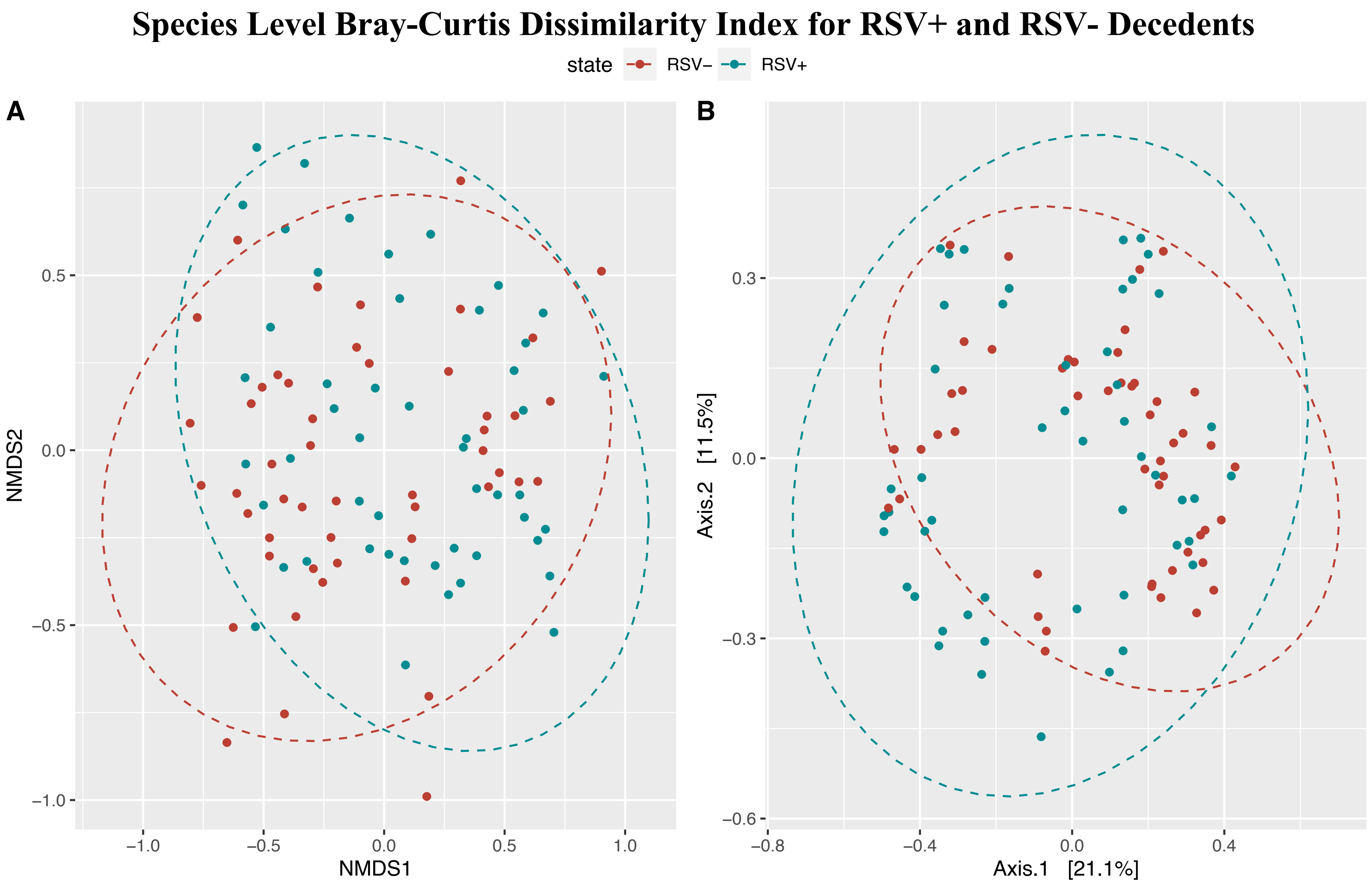

Figure, Supplemental Digital Content 3. Species analyses displaying the Bray Curtis dissimilarity index between RSV+ and RSV- infants. **A)** NMDS overlap between RSV+ and RSV- samples with a stress value of 0.262 showing very little distinct clustering. **B)** PCoA indicating some unique clustering of RSV+ in bottom left-hand corner.
