# Supplementary material for: Post-mortem Nasopharyngeal Microbiome Analysis of Zambian Infants With and Without Respiratory Syncytial Virus Disease: A Nested Case Control Study
Source: Pediatr Infect Dis J. Author manuscript; Available in PMC 2023 Sep 27. (PMC10348642; doi:10.1097/INF.0000000000003941)
Supplement: Supplemental Digital Content 1 [file NIHMS1888374-supplement-Supplemental_Digital_Content_1.pdf]

Text, Supplemental Digital Content 1.

MATERIALS AND METHODS

Study Population and Study Design

The ZPRIME study systematically enrolled 2,286 infants that died at ages ranging from four days to six months in Lusaka, Zambia between 2017 and 2020, omitting a 6-week window in 2020 due to restrictions as a result of COVID-19. Sampling for our study occurred on December 10, 2019 and thus only includes decedents between 2017 and 2019. Decedents were enrolled within 48 hours of death and, for our study, were either brought in dead (BID) or died in a facility within 48 hours of hospitalization (denoted as an early facility death) which resulted in 779 community deaths for our analysis. Early facility deaths were included as community deaths since these decedents would likely not be affected by nosocomial exposure due to limited time in the hospital. Study activities were concentrated at the University Teaching Hospital and associated morgue in Lusaka. For early facility deaths, these data were abstracted from medical chart records and official death certificates. For BID infants, we interviewed the decedents' next of kin and collected a verbal autopsy that focused on identifying whether the infant had a respiratory illness as the cause of death. Selection bias may be present since our study only focused on deaths in the morgue associated with the University Teaching Hospital where over 80% of all deceased individuals in Lusaka pass through. Some community deaths will not be captured in our study population and thus cannot be selected for participation.

Three study clinicians reviewed the verbal autopsy or clinical information available for each death to determine if the cause of death was respiratory, non-respiratory, or uncertain. Discordant adjudications were discussed among the three clinicians and either resolved or labelled as uncertain if agreement could not be reached. The clinicians were blinded to the laboratory results during this process.

The ZPRIME study had a total of 2,286 decedents, of which 779 community deaths with RSV PCR results were available at the time of this analysis. We limited our study to infants that were either BID or early facility deaths marked as RSV+ with a PCR Ct<35, resulting in 62 decedents. We upheld a more stringent RSV cut off in our criteria to exclude incorrect RSV diagnoses within our study, thereby increasing specificity. 16 decedents were excluded from our study as they did not meet this RSV PCR threshold. Additionally, we considered infants who died within 48 hours of admittance to a facility as a community death as it is unlikely that their microbiome would have been affected by nosocomial exposure. However, antibiotic usage was not known for any of the deceased infants included in this study. The 62 RSV+ decedents were matched to 62 RSV- decedents and then a random sample of 50 pairs was selected for our analysis.

Sample Collection, Processing, and Storage

All NP samples were collected from decedents at the time of enrollment into ZPRIME. NP samples were obtained using flocked-tipped nylon swabs (Copan Diagnostics, Murrieta CA) sized appropriately for infants. Two swabs were used per decedent. Each swab was inserted into either the right or left nostril until contacting the posterior nasopharynx, then rotated 180 degrees to the

left and then to the right. The swabs were then placed in 3mL of universal transport media at 2–8°C, put on ice, and transferred within the hour of collection to an onsite lab on the same campus (University Teaching Hospital in Lusaka, Zambia), where they were aliquoted and stored at -80°C until DNA extraction. DNA was extracted using the NucliSENS EasyMagG system (bioMerieux, Marcy, l'Etoile, France,) and was stored at the University Teaching Hospital at -80°C. Extracted DNA was stored at the lab located at the University Teaching Hospital in Lusaka at -80°C. Sample collection, processing and storage was previously described in Gill et al.[1]

16S rDNA Amplification and Sequencing

16S ribosomal DNA was amplified using PCR with primers specific to the V3–V4 region. PCR products and negative controls were visualized, the indexed libraries were purified and quantified, and sequenced as previously described in Lapidot et al, including control for contamination.[2,3] Negative controls were confirmed negative with no observed band on Agilent TapeStaion. Negative controls were also sequenced which resulted in extremely low reads and thus were not further analyzed. Samples were sequenced on the Illumina MiSeq sequencing platform at the National Institute for Communicable Diseases, Sequencing Core Facility (South Africa) using a 2x300 cycle V3 kit, following standard Illumina sequencing protocols.

Data Processing

We used FastQC v0.11.9 to assess the quality of reads; our multiQC report is available on Github (https://github.com/jessmcc22/ZPRIME_RSV/blob/main/multiqc_report.html).[4] All reads (both forward and reverse) had a mean Phred score > 30 (> 99.9% accuracy), indicating a good sequence quality for all the samples included in this analysis. Illumina adapters were trimmed and low quality sequences were removed using Trimmomatic v.0.39 with the following parameters: SLIDINGWINDOW: 4:20, LEADING:3, TRAILING:3, and MINLEN:36.[5] We used PathoScope 2.0 with its default parameters to quantify the proportions of reads from individual microbial strains present in the metagenomic sequencing data.[6,7] We used Refseq bacterial and viral genomes which were downloaded on November 2, 2018 as PathoScope reference libraries.[8] We have made these available in our GitHub repository (https://github.com/jessmcc22/ZPRIME_RSV#readme). Analysis was completed at both the genus and species level, which has been shown to be possible using Pathoscope.[9] PICRUSt2 was used to infer pathway abundances based on the PathoScope output.[10–15]

All raw data, processed data, and code used for data preprocessing, statistical analysis, and figure generation is available on GitHub (https://github.com/jessmcc22/ZPRIME_RSV). Raw data is also available on the sequence read archive, accession number PRJNA913857. All data and statistical analysis were completed in R version 4.2.0, primarily using packages ALDEx2 v. 1.28.1, animalcules 1.12.0, ggplot2 3.3.6, ggpubr 0.4.0, paletteer 1.5.0, phyloseq 1.40.0, and tidyverse 1.3.1.[16–25] Animalcules was used for initial exploratory visualization of the data and to create a Multi-Assay Experiment object from the raw data reads. From this object, a Phyloseq object was created and this Phyloseq object was used for statistical analysis moving forward, including creating the figures in this paper. ALDEx2 was used to analyze the PICRUSt2 results using Wilcoxon rank sum test and to visualize results with various plotting functions. All other packages were used for data manipulation and visualization.

RESULTS

Pathway Analysis

Using PICRUSt2, we inferred pathway abundances for each sample based on the microbial abundances computed with PathoScope.[6,7,9,10] We analyzed these abundances using a Wilcoxon rank sum test to determine which pathways were differentially present between RSV+ and RSV- decedents. This provided 20 different pathways; of these, 3 pathways were known to be correlated with Haemophilus influenzae (See Table, Supplemental Digital Content 5). Of the remaining 17 pathways, 10 were correlated with E. Coli, 4 correlated to a variety of plant species, and 3 correlated to other infectious diseases (Mycobacterium tuberculosis and various species of Chlamydia).

References

1. Gill CJ, Mwananyanda L, MacLeod W, Kwenda G, Mwale M, Williams AL, Siazeele K, Yang Z, Mwansa J, Thea DM. Incidence of severe and nonsevere pertussis among HIV-exposed and-unexposed zambian infants through 14weeks of age: Results from the southern Africa mother infant pertussis study (samips), a longitudinal birth cohort study. Clinical Infectious Diseases. 2016;63.

2. Klindworth A, Pruesse E, Schweer T, Peplies J, Quast C, Horn M, Glöckner FO. Evaluation of general 16S ribosomal RNA gene PCR primers for classical and next-generation sequencing-based diversity studies. Nucleic Acids Res. 2013;41(1).

3. Lapidot R, Faits T, Ismail A, Allam M, Khumalo Z, MacLeod W, Kwenda G, Mupila Z, Nakazwe R, Segrè D, Johnson WE, Thea DM, Mwananyanda L, Gill CJ. Nasopharyngeal Dysbiosis Precedes the Development of Lower Respiratory Tract Infections in Young Infants, a Longitudinal Infant Cohort Study. Gates Open Res. F1000 Research Ltd; 2022 Apr 12;6:48.

4. Andrews S. FastQC - A quality control tool for high throughput sequence data. http://www.bioinformatics.babraham.ac.uk/projects/fastqc/. Babraham Bioinformatics. 2010;

5. Bolger AM, Lohse M, Usadel B. Trimmomatic: A flexible trimmer for Illumina sequence data. Bioinformatics. 2014;30(15).

6. Hong C, Manimaran S, Shen Y, Perez-Rogers JF, Byrd AL, Castro-Nallar E, Crandall KA, Johnson WE. PathoScope 2.0: A complete computational framework for strain identification in environmental or clinical sequencing samples. Microbiome. 2014;2(1).

7. Byrd AL, Perez-Rogers JF, Manimaran S, Castro-Nallar E, Toma I, McCaffrey T, Siegel M, Benson G, Crandall KA, Johnson WE. Clinical PathoScope: Rapid alignment and filtration for accurate pathogen identification in clinical samples using unassembled sequencing data. BMC Bioinformatics. 2014;15(1).

8. O’Leary NA, Wright MW, Brister JR, Ciufo S, Haddad D, McVeigh R, Rajput B, Robbertse B, Smith-White B, Ako-Adjei D, Astashyn A, Badretdin A, Bao Y, Blinkova O, Brover V, Chetvernin V, Choi J, Cox E, Ermolaeva O, Farrell CM, Goldfarb T, Gupta T, Haft D, Hatcher E, Hlavina W, Joardar VS, Kodali VK, Li W, Maglott D, Masterson P, McGarvey KM, Murphy MR, O’Neill K, Pujar S, Rangwala SH, Rausch D, Riddick LD, Schoch C, Shkeda A, Storz SS, Sun H, Thibaud-Nissen F, Tolstoy I, Tully RE, Vatsan AR, Wallin C, Webb D, Wu W, Landrum MJ, Kimchi A, Tatusova T, DiCuccio M, Kitts P, Murphy TD, Pruitt KD. Reference sequence (RefSeq) database at NCBI: Current status, taxonomic expansion, and functional annotation. Nucleic Acids Res. 2016;44(D1).

9. Faits T, Odom-Mabey AR, Castro-Nallar E, Crandall KA, Johnson WE. Metagenomic profiling pipelines improve taxonomic classification for 16S amplicon sequencing data. bioRxiv [Internet]. 2022 Jan 1;2022.07.27.501757. Available from: http://biorxiv.org/content/early/2022/07/29/2022.07.27.501757.abstract

10. Douglas GM, Maffei VJ, Zaneveld JR, Yurgel SN, Brown JR, Taylor CM, Huttenhower C, Langille MGI. PICRUSt2 for prediction of metagenome functions. Nature Biotechnology. 2020.

11. Barbera P, Kozlov AM, Czech L, Morel B, Darriba D, Flouri T, Stamatakis A. EPA-ng: Massively Parallel Evolutionary Placement of Genetic Sequences. Systematic Biology. 2019.

12. Czech L, Barbera P, Stamatakis A. Genesis and Gappa: Processing, analyzing and visualizing phylogenetic (placement) data. Bioinformatics. 2020;36(10).

13. Mirarab S, Nguyen N, Warnow T. SEPP: SATé-enabled phylogenetic placement. Pacific Symposium on Biocomputing. 2012.

14. Louca S, Doebeli M. Efficient comparative phylogenetics on large trees. Bioinformatics. 2018;34(6).

15. Ye Y, Doak TG. A parsimony approach to biological pathway reconstruction/inference for genomes and metagenomes. PLoS Comput Biol. 2009;5(8).

16. Team RC. R: A Language and Environment for Statistical Computing. R Foundation for Statistical Computing. 2021.

17. Zhao Y, Federico A, Faits T, Manimaran S, Segrè D, Monti S, Johnson WE. animalcules: interactive microbiome analytics and visualization in R. Microbiome. 2021;9(1).

18. McMurdie PJ, Holmes S. Phyloseq: An R Package for Reproducible Interactive Analysis and Graphics of Microbiome Census Data. PLoS One. 2013;8(4).

19. Wickham H. Package `ggplot2`: Elegant Graphics for Data Analysis. Springer-Verlag New York. 2016;

20. Kassambara A. ggpubr: "ggplot2" based publication ready plots. R package version 0.2. https://CRANR-project.org/package=ggpubr. 2020;

21. Hvitfeldt E. paletteer: Comprehensive Collection of Color Palettes. 2021.

22. Wickham H, Averick M, Bryan J, Chang W, McGowan L, François R, Grolemund G, Hayes A, Henry L, Hester J, Kuhn M, Pedersen T, Miller E, Bache S, Müller K, Ooms J, Robinson D, Seidel D, Spinu V, Takahashi K, Vaughan D, Wilke C, Woo K, Yutani H. Welcome to the Tidyverse. J Open Source Softw. 2019;4(43).

23. Fernandes AD, Macklaim JM, Linn TG, Reid G, Gloor GB. ANOVA-Like Differential Expression (ALDEx) Analysis for Mixed Population RNA-Seq. PLoS One. 2013;8(7).

24. Fernandes AD, Reid JN, Macklaim JM, McMurrough TA, Edgell DR, Gloor GB. Unifying the analysis of high-throughput sequencing datasets: characterizing RNA-seq, 16S rRNA gene sequencing and selective growth experiments by compositional data analysis. Microbiome. 2014 Dec 5;2(1):15.

25. Gloor GB, Macklaim JM, Fernandes AD. Displaying Variation in Large Datasets: Plotting a Visual Summary of Effect Sizes. Journal of Computational and Graphical Statistics. 2016;25(3).
